# Does social context impact metacognition? Evidence from stereotype threat in a visual search task

**DOI:** 10.1371/journal.pone.0215050

**Published:** 2019-04-15

**Authors:** Thibault Gajdos, Isabelle Régner, Pascal Huguet, Marine Hainguerlot, Jean-Christophe Vergnaud, Jérôme Sackur, Vincent de Gardelle

**Affiliations:** 1 Aix Marseille Univ, CNRS, LPC, Marseille, France; 2 Université Clermont Auvergne, CNRS, LAPSCO, Clermont-Ferrand, France; 3 Centre d’Economie de la Sorbonne, CNRS UMR 8174, Paris, France; 4 Laboratoire de Sciences Cognitives et Psycholinguistique (ENS, CNRS, EHESS), PSL Research University, Paris, France; 5 Ecole Polytechnique, Palaiseau, France; 6 CNRS and Paris School of Economics, Paris, France; Macquarie University, AUSTRALIA

## Abstract

While recent studies have emphasized the role of metacognitive judgments in social interactions, whether social context might reciprocally impact individuals’ metacognition remains an open question. It has been proposed that such might be the case in situations involving stereotype threat. Here, we provide the first empirical test of this hypothesis. Using a visual search task, we asked participants, on a trial-by-trial basis, to monitor the unfolding and accuracy of their search processes, and we developed a computational model to measure the accuracy of their metacognition. Results indicated that stereotype threat enhanced metacognitive monitoring of both outcomes and processes. Our study thus shows that social context can actually affect metacognition.

## Introduction

Metacognition, i.e., the process of monitoring and controlling one’s own cognitive processes [1], plays a crucial role in the regulation of our behavior [2]. It might be either implicit, involving automatic cognitive processes, or explicit, relying on conscious reflection. Recent research demonstrated the importance of both forms of metacognition in social interactions (see [3], for a review). For instance, it has been shown that, even in a simple visual task, dyads perform better than each member separately, when their members can share their confidence about their visual perceptions [4].

It has also been shown that social cues within the task might affect individuals’ metacognition [5,6], however, whether social context might impact individuals’ metacognitive processing is, to the best of our knowledge, an open question. We argue that candidates to investigate this question are social contexts involving stereotype threat, i.e. situations in which individuals feel themselves to be at risk of confirming a negative stereotype about their social group [7]. First, as detailed below, stereotype threat is a ubiquitous social situation, and its study is one of the most active fields in social psychology [8]. Second, and more importantly, it has been hypothesized that stereotype threat might increase the allocation of attention to internal processes such as metacognition [9]. We report here an experiment based on a visual search task, in which we directly tested this hypothesis.

### Stereotype threat

In a celebrated article, Steele and Aronson [7] demonstrated that stereotype threat occurs when individuals find themselves in a situation where individuals can feel the threat of confirming a negative stereotype that could provide a plausible explanation for their performance. One explanation offered for the effect of stereotype threat is that it leads to a physiological stress response and negative thoughts that diminish the cognitive resources (e.g. attention, working memory) needed for successful performance on complex tasks [10,11]. Stereotype threat is thus likely to result in impaired performance when one cannot compensate for the depleted cognitive resources such as when the test is difficult. This has been shown to be the case across populations and domains like women on standard math tests [12,13], boys on reading tests [14], low-socio-economic background students and ethnic minorities on intellectual tests [15,16], older adults on memory tests [17,18] or white men on athletic tests [19]. It should be noted that although stereotype threat also induces a disruptive evaluative pressure on easier tests, it is less likely to result in decreased performance on such tests because individuals can compensate for the depletion of their cognitive resources by expending more effort [12,20,21].

A number of studies have also described several factors that modulate the effects of stereotype threat on performance. First, as mentioned above, these effects are more pronounced when the task is challenging, that is, when it is difficult and thus induces doubts in the eyes of the individuals themselves as to the quality of their performance. Second, stereotype threat has also been shown to be stronger in individuals who highly *identify* to the domain, i.e. individuals who care about their performance in the task, who believe that performing well is important and who consider that they have much to lose in the event of poor performance [14,22,23]. Finally, stereotype threat effects seem also stronger when the task drives attention to gains rather than losses [24,25], which has been interpreted under the Regulatory Focus Theory [26]. Under this account, it is argued that stereotype threat leads the individual to adopt a prevention focus where he or she tries to avoid losses, while performance in the task is usually associated with a promotion focus where attention is directed towards gains. This mismatch between prevention and promotion is thought to reduce the participant’s willingness to engage in the task, thereby deteriorating his/her performance [24,25].

### Stereotype threat and metacognition

The mechanisms underlying stereotype threat have also come under consideration [27,28]. It has been proposed that stereotype threat might induce physiological stress and efforts to suppress negative thoughts, thereby taxing working memory resources typically required for successful performance on difficult tasks [13,29]. Another possible explanation involves implicit metacognition: stereotype threat might increase individuals’ uncertainty about their abilities, leading to increased attention to their own behavior and performance. This increased attention might disrupt task performance by interfering with mental processes that usually run automatically [30], such as in proceduralized motor tasks, or by taxing cognitive resources needed to complete difficult, conceptual tasks [10,29]. However, if attention towards internal states increased, one should also predict a positive consequence of stereotype threat: individuals should be better at evaluating their own decisions and the cognitive processes involved in task performance under stereotype threat. In other words, if it increases attention towards internal states, it is likely that stereotype threat will also improve explicit metacognition (e.g. confidence judgments). According to this view, stereotype threat would have a paradoxical effect, of improving the quality of self-monitoring, at the cost of hindering the performance in the task execution. When the task is difficult, this paradoxical effect might come from a competition for cognitive resources needed to the execution of the task on one hand and monitoring on the other hand. In the case of routine (proceduralized) tasks, an increase in self-monitoring might induce a switch from efficient and automatic processes to deliberated, controlled and less efficient processes.

Several studies suggested that stereotype threat enhances implicit metacognition during task performance. For instance, one study showed that women under stereotype threat are more likely to correct erroneous responses on an antisaccade task [31]. Another study [32] using EEG in a conflict task, found that the amplitude of the fronto-medial negativity–which typically appears after errors [33,34]–was increased under stereotype threat, a finding interpreted as reflecting an increased vigilance towards errors under stereotype threat. A subsequent study [21] reported that in a Stroop task the fronto-medial response associated with conflicts and errors was larger for women under stereotype threat in no-conflict trials; this finding was interpreted as a sign of over-responsiveness of the conflict monitoring system under stereotype threat.

However, none of the studies described above measured explicit metacognition. Moreover, whether stereotype threat improves metacognition about the internal processing steps involved in task performance (and not only metacognition about the outcome of task performance) remains an open empirical question. The present study aims at filling this gap, by directly testing whether stereotype threat yields better explicit monitoring of cognitive processes on the one hand, and better post-evaluation of decisions on the other.

### Stereotype threat and metacognition during visual search

Typically, assessing metacognitive monitoring is done by asking subjects to report their subjective confidence after a decision. Other measures of introspection exist, however, such as judgments about the task duration [35] or the visibility of a stimulus [36], depending on the particular task at hand. In a visual search task, where participants have to find a target element embedded in a set of distractors (e.g. find a X amongst a set of Ts), they might be asked about the number of items that they have inspected before noticing the search target, or in other words the subjective number of scanned items (hereafter SNSI) [37]. Typically, finding a target L in a set of Ts takes more time when there are more Ts, but finding a X does not, and participants can acknowledge this fact. If in addition they have to evaluate another feature of the target (e.g. was the X green or red?), participants can also indicate their confidence in this decision.

Here, we used this visual search task, following Reyes and Sackur [37]. Subjects searched a set of items for a target and had to report the color of the target. After each trial they were asked about their confidence in their response and the subjective number of items scanned during the search process. We computed two measures of metacognitive monitoring. The first one is the Brier Score, which characterizes the overall mismatch between confidence and judgment accuracy [38]. The second measure is both novel and independent from the first. It quantifies the ability of participants to introspect the search process, by measuring the absolute difference between the reported SNSI and the actual number of scanned items, which we estimated on the basis of a simple computational model of visual search [39–41].

This paradigm allowed us to evaluate the impact of stereotype threat on metacognitive monitoring. Specifically, males and females students in science performed the task, which was presented as relying on visuo-spatial ability, a domain where women are typically targeted by a negative gender stereotype. To strengthen this idea of a potential threat “in the air” for women on the visual search task, participants were also instructed that this task was also predictive of geometry ability (which may be especially important for students in science) or ability to read a map. Previous research demonstrated that instructions relating the focal task to visuo-spatial abilities [42] and/or geometry ability [43,44] were very efficient to induce stereotype threat effects. In the threat (vs. no-threat) condition, participants were informed that previous studies found a difference (vs. no difference) between men and women performance on that task. Because it has been shown that stereotype threat is stronger among individuals who strongly identify to the task domain [14,22,23], we measured whether participants did identify with the visuo-spatial abilities involved in the task. Moreover, since it is also stronger when the task involves gains rather than losses [24,25], we also manipulated the gain versus loss framing of the task: one half of the trials were presented in a gain frame (participants received two points for each correct response), the other half in a loss frame (one point earned for each correct response, 3 points lost for each incorrect response).

Our main measures of interest are about metacognitive abilities (i.e. the Brier Score and the SNSI error, see Methods). In particular, we expected that stereotype threat would produce its strongest effects on metacognition in the gain frame, for women who highly identify with the task domain. Performance in the visual search task was also a measure of interest, but our ability to observe the negative effects of stereotype threat on performance is not guaranteed, since previous studies have also found that the effect of stereotype threat on performance can vanish when the test is too easy [12,22].

## Method

### Participants

Participants were 125 students in sciences form Aix-Marseille University. This sample size was limited by time and budget constraints only, and consistent with previous studies. Each participant received compensation of €10. Participants were randomly assigned to the threat condition (31 women, 31 men) or the no-threat condition (31 women, 32 men). All participants reported normal or corrected to normal vision.

### Ethics statement

Written informed consent was obtained from all participants before the experiment. The stereotype threat treatment was fully explained in a debriefing after the experiment. Because the research involved negligible risks and no nominative/identifying information was collected, ethics approval was not required under current French regulations, and no IRB was consulted before conducting the study.

### Stimuli and task

**Fig 1:**
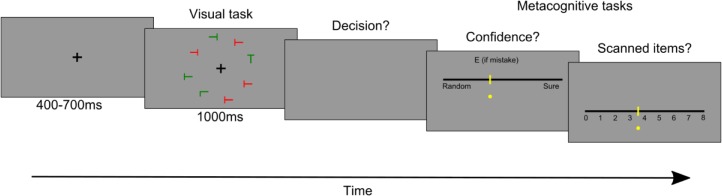
Example trial sequence. Participants have to search for a target (L or X) amongst distractors (T) and report its color. Then, they indicate their confidence in this decision and the number of items scanned during the search process.

The experimental paradigm is very similar to the one used in experiment 3 in Reyes and Sackur [37], and involves a visual search task and two introspective scales (Fig 1).

Each trial began with a central fixation cross (random duration between 400 ms and 700 ms). An array of letters (size: 0.8×1.2°), regularly spaced on an imaginary circle (radius: 5.5°) around fixation, was then presented for 1s or until the participant’s response, whichever came first. This array contained 8, 14, or 20 letters, randomized across trials. One of these elements, randomly selected, was the letter X or the letter L (the target) and the others were Ts (distractors). The vertical/horizontal orientation of each element was randomly drawn in each trial. A random half of the letters were colored in red, the other half in green. The task of the participant was to decide whether the target was red or green, which was randomized across trials. Participants provided their response on a standard French keyboard, by pressing either the ‘D’ key (which was covered with a red sticker) with the left index, or the ‘K’ key (which was covered with a green sticker), with the right index. A red and a green sticker were also placed on the top left and top right corners of the keyboard, respectively.

Each trial was followed by two introspective reports, presented successively on visual analog scales, on which participants responded using the mouse. One scale required to estimate the number of items scanned before the identification of the target (Subjective Number of Items Scanned, SNSI), and was labelled from 0 to the set-size of the trial. The other scale required the participant to estimate his or her confidence from "guess" to "certain", with the "E" key in case of mistake. The order of the presentation of the scale was randomized across participants.

Stimuli were presented on a 24’ CRT screen (resolution of 1920×1200 pixels, refresh rate: 60Hz, distance about 55 cm), using the Psychophysics Toolbox [45] in MATLAB (the MathWorks, Natick, MA).

### Procedure

Participants met individually one of the two female research assistants, who explained thoroughly the protocol. In particular, participants were told that the visual search task they would complete was predictive of visuo-spatial abilities, and was also related to geometry ability or ability to read a map. In the threat condition, the experimenter furthermore explained that previous studies had found a difference between male and females’ performances in the visual search task. By contrast, in the no-threat condition, it was stated that previous studies found no differences between men and women. This control, no-threat condition takes into account the fact that the lack of gender information does not necessarily prevent stereotype threat effects, the stereotype is implicitly activated by the task itself whenever it falls into a domain where a negative stereotype is relevant. Thus, a true control condition implies either a characterization of the test as non-diagnostic of the ability targeted by the stereotype [43,44,46] or a verbal falsification of the stereotype by using what is typically called “gender fair instructions” [12,13] such as those used here. The exact instructions given to participants in both conditions (threat vs. no-threat) can be found in the supplementary material (S1 File).

Participants then sat at the computer. Three instruction screens reinstated the alleged aim of the study, described the perceptual task. Participants were then asked to specify their field of study. In the stereotype threat condition, they were furthermore asked to indicate their sex.

The experiment was divided in three blocks. Participants had to call the experimenter at the beginning of each block, at which point the experimenter provided again detailed explanations concerning the forthcoming block. The first block was a training phase, consisting in 24 trials (12 slow trials with a 2s duration, and 12 trials at normal speed). Then came the two experimental blocks: one in a gain frame (subjects received two points for each correct response), and the other one in a loss frame (subjects received one point for each correct response, but lost 3 points for each incorrect response). Half of the participants received the gain frame first, and half received the loss frame first. Each of these blocks contained 180 trials (15 trials with each target color, target type and set-size condition, randomized within blocks).

Finally, because the visual search task was presented to the participants as assessing visual spatial abilities—a domain where women are typically targeted by a negative gender stereotype—we measured how much they identified with these abilities (assuming that higher identification leads to higher stereotype threat, [14,22,23]. These questions were: "Q1: for you, it is important to succeed in that kind of test measuring visuo-spatial abilities" and "Q2: the ability to represent objects in space is important in your life in general", "Q3: for you, the ability to orient yourself in space is important", "Q4: the ability to represent objects in space is important in your studies". The order of these questions was the same for all participants. The responses were given on analogical scales with gradations ranging from: "not important at all" to "very important".

### Performance measures

Performance in the visual search task is measured by accuracy and response time. We checked whether stereotype threat impact any of these measures.

### Brier score

Confidence was reported on an analogical scale from "guess" to "certain", which we converted into a numerical scale ranging from 0 (corresponding to "guess") to 1 (corresponding to "certain"). We excluded trials that participants identified as mistakes by pressing the "E" key when providing their confidence rating. These corresponded to less than 4.8% of the trials, and we ensured that all our results are qualitatively similar when we exclude from the analysis participants who used this option for more than 10% of the trials.

Confidence was strongly right-skewed: the "certain" response was used for 65% of the trials, and the average confidence was 87% overall. The median confidence was equal to "certain" for 100 participants out of 125. To compensate for this skewness and enhance the sensitivity of our confidence data, we redefined our confidence as a binary variable by coding trials with confidence equal or greater than the individual median as 1 and trials with lower confidence as 0. From this binarized confidence, for each participant we computed a Brier score [38], which is defined as the average of the quadratic difference between trial accuracy and confidence (defined as described above). It is therefore also a measure of the quality of confidence. Smaller Brier scores indicate that confidence judgments are better aligned with performance.

### SNSI error

SNSI error was assessed through a simple computational model of guided visual search in the spirit of previous studies [40,41], which build on Luce’s celebrated model [39]. The basic idea is that the probability to look directly at a target t (where t = "X" or t = "L") in frame f is simply the salience *w* of the target, relative to the salience of the whole set. Normalizing (without loss of generality), the salience of the distractors to one, the salience of the whole set is thus equal to *w+n-*1, where *n* is the number of elements in the display. As a result, and noting that the relative salience might depend on the type of target and on the frame, the probability to detect a target t in frame f is given by:

$$P(t,f,n)=\frac{w(t,f)}{w(t,f)+n-1}$$ (1)

The search process is assumed to be sequential: the subject pick and inspect a first item. If this item is the target (which happens with probability *P*(*t*,*f*,*n*)), the search process ends. Otherwise (with probability 1-*P*(*t*,*f*,*n*)), the subject eliminates the inspected item, and selects a new item among the (*n*-1) remaining ones. Let *N*(*t*,*f*,*n*) be the expected number of inspected items until a decision is made, when facing a set of *n* items. By the above reasoning,

N(t,f,n)=P(t,f,n)+(1−P(t,f,n))N(t,f,n−1) (2)

Of course, if there is no distractor (i.e., *n* = 1), the target will be detected with certainty at the first inspection, i.e., *N*(*t*,*f*,1) = 1. Given this initial condition, one can solve the difference Eq (2) to get:

$$N(t,f,n)=\frac{w(t,f)+n}{w(t,f)+1}$$ (3)

We actually observe neither *N*(*t*,*f*,*n*) nor *w*(*t*,*f*). What is available is the reported number of inspected items, *Ñ*(*t*,*f*,*n*). Here, we assume that this reported number might be equal to the actual number of inspected items, plus a bias noted *β*(*t*,*f*) that does not depend on the number of items *n*. We thus have:

$$\beta (t,f)=N(t,f,n)-\tilde{N}(t,f,n)$$ (4)

The SNSI error is then given by the absolute value of *β*(*t*,*f*). Eq (4) implies, for any numbers of items *n* and *m*, we have:

$$\tilde{N}(t,f,n)-\tilde{N}(t,f,m)=N(t,f,n)-N(t,f,m)$$ (5)

Besides, from Eq (3) we have:

$$N(t,f,n)-N(t,fm)=\frac{n-m}{w(t,f)+1}$$ (6)

Thus, given Eq (5) and Eq (6):

$$\tilde{N}(t,f,n)-\tilde{N}(t,f,m)=\frac{n-m}{w(t,f)+1}$$ (7)

We can now isolate *w* in Eq (7), and substitute it in Eq (3), which leads to:

$$N(t,f,n)=\frac{n-m+(n-1)(\tilde{N}(t,f,n)-\tilde{N}(t,f,m))}{\tilde{N}(t,f,n)-\tilde{N}(t,f,m)}$$ (8)

Finally, Eqs (8) and (4) imply:

$$\beta (t,f)=\frac{\left(\tilde{N}(t,f,n)-1)(n-m)-(n-1)(\tilde{N}(t,f,n)-\tilde{N}(t,f,m)\right)}{n-m}$$ (9)

The absolute value of *β*(*t*,*f*) is the measure of interest here. Under the assumptions of our model, it quantifies how much participants misestimate the number of items they have inspected during their search process. Note that Eq (9) holds for any pairs of numbers of displayed items *n* and *m*. In our data we noticed that the number of scanned items reported for 14 items was often lower than for 8 items or higher than 20 items, which seems implausible. We therefore used *n* = 8 and *m* = 20 in our dataset to compute the SNSI error.

### Identification to visual spatial abilities

A reliability analysis was conducted on the four identification items (using the *alpha* function of the *psych* package in R). Cronbach’s alpha for the whole scale was relatively low (0.57) and varied from 0.44, 0.58, 0.52 or 0.44 respectively when Q1, Q2, Q3 or Q4 were removed. This analysis indicated that the two questions most contributing to the scale were Q1 and Q4. Thus, we used Q1 and Q4 averaged for measuring participant’s identification to visuo-spatial abilities. Within each of the 4 sex-treatment groups, we then defined highly identified subjects as those with an identification score above the median of the group. This identification variable was constructed independently of the data analysis.

### Statistical tests

All outcomes were analyzed with linear mixed models using the *lmer4* package [47] in R (version 3.3.1 [48]). All regressions were performed with the restricted maximum likelihood fitting method, and p values for coefficients were obtained with the *car* package [49]. Means and 95% confidence intervals were computed using a bootstrap procedure implemented with the *boot* package [50]. Finally, post hoc comparisons used permutation tests [51], with p values Bonferonni corrected for the two metacognitive measures. We used an alpha level of .05 for all statistical tests.

## Results

All outcomes were primarily analyzed through generalized hierarchical linear mixed-effects regressions with target type (either X or L), the number of displayed items (set size) and their interactions and treatment (i.e. threat vs. no-threat), sex, frame (gain vs. loss) and identification (high vs. low) and their interactions as fixed effects. The model thus contains the intercept, the effect of target type, set-size and their interactions, as well as the frame, as random-effects. We focus on the effects of interest in the main text of the manuscript, and in particular on the four-way interaction involving sex, stereotype threat treatment, frame and identification. The full tables of the regression results are presented in the supplementary material.

### Performance

Response accuracy in the visual search task was high overall (M = 88%, SD = 7%, see also Table A in S1 File). As expected, it was affected by the parameters of the stimuli: participants were less accurate when searching for an "L" than for an "X" among "Ts" (p < 0.001), accuracy decreased with the number of distractors (p < 0.001), and these two effects interacted (p < 0.001). We found no evidence of a significant effect of stereotype threat on response accuracy: all interactions involving sex and stereotype threat were not significant (all ps > 0.6). The full table of the results of the linear mixed model is presented in the supplementary material (Table B in S1 File).

Response times showed a similar pattern (Table C in S1 File). The average median response time on correct trials was 1.18s (SD = 0.421s). As expected, searching for an "L" takes more time than searching for an "X" among "Ts" (p < 0.001), response time increased with the number of distractors (p < 0.001), and these two variables interacted (p < 0.001). We found no evidence of a significant effect of stereotype threat on response times: all interactions involving sex and stereotype threat were not significant (all ps > 0.6).

In sum, performance in the visual search task, as assessed by response accuracy and response times, was affected by the parameters of the stimuli (target type and set size), but not by the stereotype threat context in which the task was performed.

### Brier score

The Brier score quantifies the total mismatch between confidence and accuracy [38], and was used as a measure of metacognition accuracy. The results of the regression (Table D in S1 File) showed that the Brier score was affected by target type (p < 0.001) and set-sizes (p < 0.001), and that these two variables interacted (p < 0.001). Brier scores were on average higher (indicating that metacognition was worse) for "L" targets and for larger set sizes.

**Fig 2:**
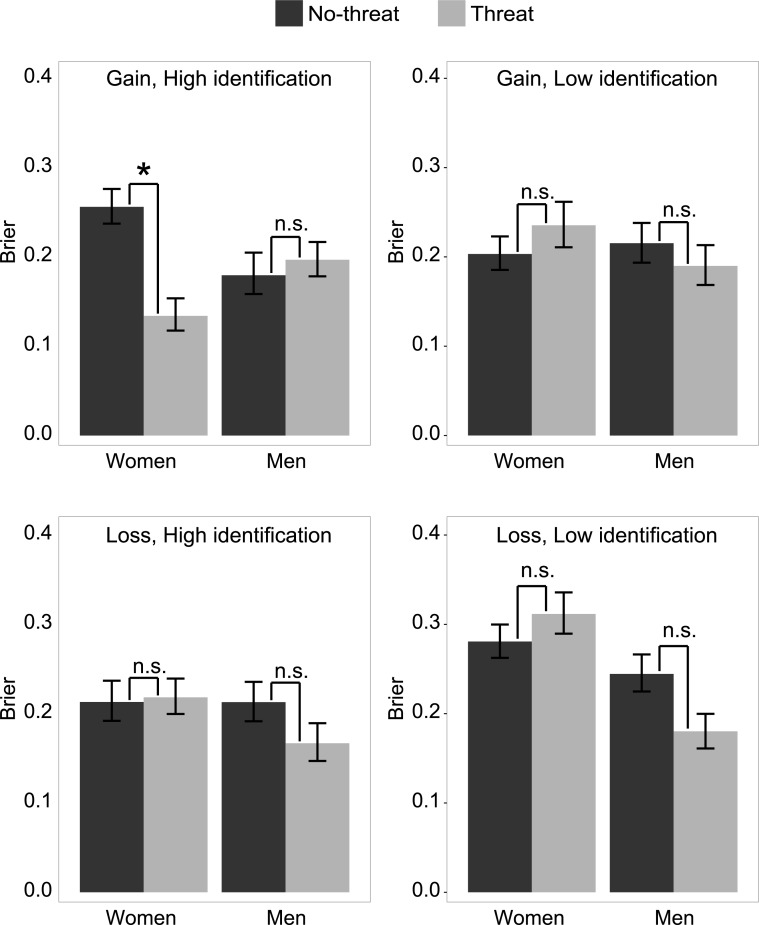
Brier scores. Mean Brier scores across participants, as a function of treatment (threat vs. no-threat), sex, frame (gain vs. loss) and identification (high vs. low). Error bars reflect 95% CI intervals. *: p < .05 (corrected for 2 comparisons); ns: p > 0.25.

Importantly, regarding the main focus of our study, we found a significant interaction between participants’ sex and identification with the task, stereotype treatment, and framing (p = 0.041), as expected. Further analyses confirmed that, as expected, the only significant effect of stereotype threat was found for women with high identification to visual-spatial abilities in the gain frame (Fig 2). Specifically, women under stereotype threat had a better metacognitive accuracy as indexed by a lower Brier score (M = 0.13, 95% CI = [0.12,0.15]) than women in the no-threat treatment (M = 0.26, 95% CI = [0.24,0.28]), p = 0.016. In all other cases, the experimental treatment of stereotype did not affect the Brier score (all ps > 0.4).

### SNSI error

Our second measure of metacognitive monitoring was based on the absolute mismatch between the theoretical and empirical subjective number of scanned items during the visual search. The results of the regression for this variable (Table E in S1 File) indicated a significant effect of target type. On average, the SNSI error was greater when the target was an "L" (*p <* 0.001).

**Fig 3:**
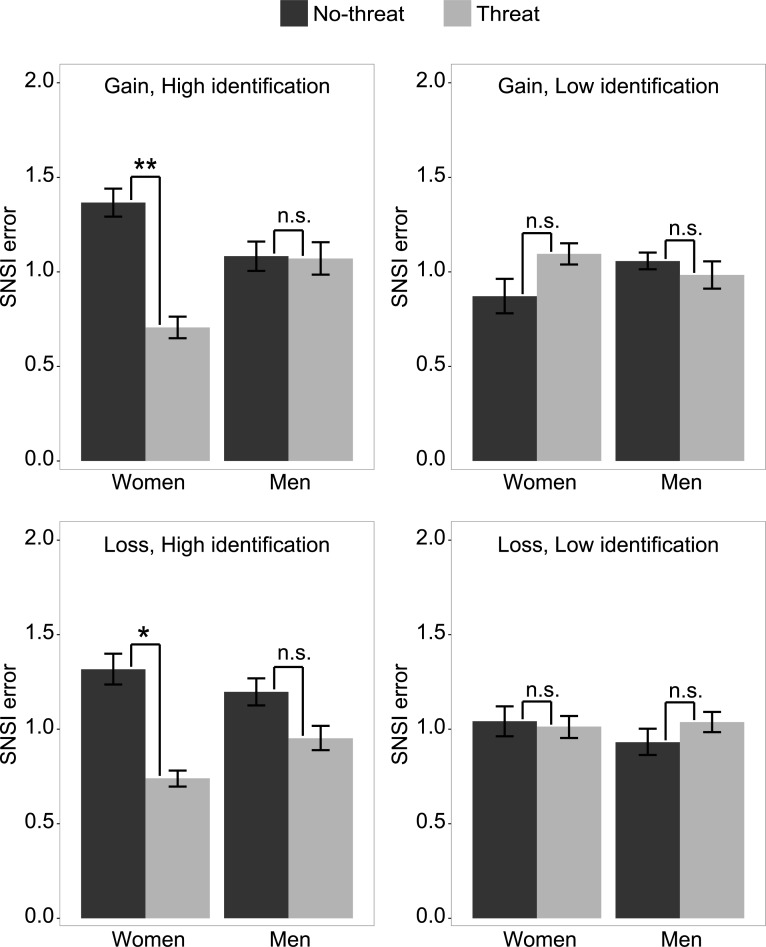
SNSI errors. Mean SNSI errors across participants, as a function of treatment (threat vs. no-threat), sex, frame (gain vs. loss) and identification (high vs. low). Error bars reflect 95% CI intervals. *: *p <* .05, **: *p <* .01 (corrected for 2 comparisons); ns: *p >* 0.25.

Crucially, we also found a significant interaction between participants’ sex and identification with the task, stereotype threat treatment, and framing (*p* = 0.046), as expected. Analyses within each condition (Fig 3) revealed that women with high identification to the task had a smaller SNSI error (i.e. a better metacognitive monitoring of the visual search process) in the threat condition than in the control condition, both in the gain frame (stereotype threat: *M* = 0.71, 95% *CI* = [0.65,0.76]; no-threat condition: *M* = 1.37, 95% *CI* = [1.29,1.44]), *p* = 0.005, and in the loss frame (stereotype threat: *M* = 0.74, 95% *CI* = [0.70,0.78]; no-threat condition: *M* = 1.32, 95% *CI* = [1.24,1.40]), *p* = 0.022. In all others cases, stereotype threat did not affect the SNSI error (all *ps >* 0.6).

## Discussion

The present study aimed at quantifying the effects of stereotype threat on metacognition during visual search. As expected from previous research on visual search tasks, we first found that performance decreased with the number of distractors, an effect that was more pronounced for "Ls" targets than for "Xs" [52]. Replicating these classic results enabled to build a model for the number of inspected items [40,41], and to evaluate whether participants had a good metacognitive access to this variable, by defining a new measure of metacognitive monitoring we called SNSI error. In addition, we used Brier scores to measure metacognitive monitoring based on confidence judgments. We found that stereotype threat did not affect performance, but did affect both measures of metacognitive monitoring. We will now discuss these two aspects in turn.

Performance was not impacted by stereotype threat. Although this result could be seen as a failure to manipulate the threat context in our experimental setting, one alternative explanation is simply that our task was too easy, at least relative to the tasks used in previous stereotype threat studies. Indeed, the overall success rate was very high in our task (M = 0.88, 95% CI = [0.87,0.88]), and previous studies have shown that stereotype threat does not affect performance on easy tasks [12,22]. It is possible that when the task is sufficiently easy, this allows individuals to compensate for the depletion of their cognitive resources by expending more effort [12,20,21], which they might be unable to do when they are already at their maximum. Thus, although stereotype threat did not produce a measurable effect on performance, it does not imply that stereotype threat had no effect at all in our task. Indeed, we have seen that it affects metacognitive abilities (and arguably, these effects are not confounded with task performance). Nevertheless, we acknowledge that to confirm this interpretation, further research would be needed to replicate the present results with a more difficult task.

Critically, whereas performance was unaffected by stereotype threat, our two measures of metacognitive monitoring (the Brier score and the SNSI error) were significantly impacted. Note that this was only true for women who highly identified with the abilities supposedly assessed by the task, as typically found in previous studies [14,19,22,53]. This interaction thus strengthens the interpretation that the observed differences are actually due to stereotype threat.

It is likely that our different measures of metacognition capture, at least partially, distinct aspects of metacognitive monitoring. Indeed, the SNSI error aims at quantifying participants’ ability to monitor the process of visual search while the Brier score is meant to evaluate the participants’ ability to monitor the accuracy of the decision. Importantly, these two measures rely on entirely different data: whereas the Brier score is based on decision accuracy and confidence judgments, the SNSI error is based on objective set-sizes and reported SNSI. We also note that these measures are only moderately correlated across participants (r = 0.23). Here we should also clarify that because the Brier score is an aggregate measure, it should be interpreted with caution. Indeed, it is known to be affected by the overall confidence bias of participants (i.e. underconfidence/overconfidence), and by the resolution of confidence with respect to performance (see e.g. [54]). However, these two factors were difficult to estimate in isolation: the overall bias was difficult to measure properly in our study, because we used a qualitative rating scale, and because of the generally high performance and high confidence levels in our data. The resolution was difficult to estimate because of the low number of errors.

Empirically, we found that while the Brier score only improved in the gain frame, the SNSI error improved both in the gain and in the loss condition, which further support the dissociation between the two measures. We acknowledge that this dissociation between the two measures, and in particular the finding that SNSI would also be affected in the loss frame, was not fully anticipated. In what follows, we would like to offer tentative explanations for the patterns found for our two measures. Our assumption is thus that our two measures evaluate different aspects of monitoring, the monitoring of decision accuracy on the one hand and the monitoring of the search process on the other hand. Our results suggest that stereotype threat enhances both types of monitoring, although in different ways.

Firstly, the finding that stereotype threat affects Brier scores only in the gain frame specifically suggests the implication of regulatory mismatch phenomenon, along the Regulatory Focus Theory [26,55]. According to this theory, individuals may concentrate on gains or other positive benefits of task performance (promotion focus) or on losses and costs to be avoided (prevention focus). It has been shown that stereotype threat generates a prevention focus [25], which induces a regulatory mismatch when the task has a reward structure based on gains [24], but not when the task involves a loss frame. Thus, the pattern of effects seen in our data is consistent with the notion that regulatory mismatch affects Brier scores. We should emphasize though that the precise mechanisms at work remain unclear. Regulatory mismatch has been appealed to account of lower "feelings of rightness" in the past [26]. One possible scenario that would reconcile our data with the notion of regulatory mismatch account is that such mismatch increases the attention devoted to decision outcomes, thereby increasing the accuracy of metacognitive monitoring, which leads to higher confidence levels in the present task. However, we insist that this scenario is very speculative at the moment and that further work would be needed to clarify this issue.

Secondly, the impact of stereotype threat on SNSI error suggests that it affects metacognitive monitoring through another channel that is not affected by regulatory mismatch *per se*. Indeed, since the SNSI error was reduced both in the gain and in the loss frames, a more generic effect of stereotype threat might be at play, independent from the one caused by regulatory mismatch. This effect could be mediated by an orientation of attention towards internal mental processes. In this scenario, the stereotype threat induces participants to better grasp the visual search process that unfolds within a trial, such that they become better able to realize how many elements they have focused on during this process.

Before we conclude, we would like to highlight some limitations of the present study. First, as already mentioned, the finding that our two measures of metacognition exhibited different results was unexpected. This finding is important since it suggests that stereotype threat effects are diverse and need not be explained by a single mechanism, but it needs to be confirmed by further empirical investigations. A second limitation of our approach is the absence of stereotype threat effect on performance measures (accuracy and response times). In the light of past studies, suggesting that such effects on performance would only arise for difficult tasks (i.e., requiring cognitive control), we have argued that our visual search task might have been too easy, compared with most tasks used in stereotype threat research. This task was not easy in the sense of routinized tasks that could be performed automatically (without attention). Instead it required attention but did not require as much as cognitive control as the tasks used in stereotype threat research. Finally, we note two limitations regarding our confidence data. In terms of design, we used a qualitative scale but had we used instead a quantitative scale (by which participants would express their “subjective probability of being correct”), we could have compared the subjective and objective performance, to obtain an index of overconfidence in our participants. In terms of analysis, the low number of errors in our task prevented us from evaluating the efficiency of confidence judgments, e.g. using the meta-d’ approach [56] to isolate the ability of the metacognitive system from the ability of the perceptual system. Further studies employing a more difficult task thus seem needed for this reason as well.

In sum, our data suggests that stereotype threat enhances metacognitive monitoring of both outcomes and processes. While recent studies have emphasized the role of metacognitive judgments in social interactions [4], here we have investigated the reciprocal link, and we show that social context might impact individuals’ metacognition when performing a simple task. To do so, we provided a formal model of the visual search task that allows inferring the internal variables underlying task performance. We could then evaluate metacognition by comparing these inferred variables with the reports of participants. We believe that our method and results provide new insights both for the study of metacognition and for that of stereotype threat. More generally, it opens the route for a wider agenda, investigating the impact of other social contexts (e.g., competition vs. cooperation, social facilitation, choking under pressure, etc.) on metacognition.

## Supporting information

S1 File Supplementary materials. Supplementary materials, including Tables S1 to S5 and experimental instructions for the stereotype threat and no-threat conditions.
(DOCX)

S2 File Data. Experimental data.
(CSV)

S3 File DataReadme. Details of the format of the experimental data.
(TXT)
