# Supplementary material for: Does social context impact metacognition? Evidence from stereotype threat in a visual search task
Source: PLoS One. 2019 Apr 15;14(4):e0215050. doi: 10.1371/journal.pone.0215050 (PMC6464175; doi:10.1371/journal.pone.0215050)
Supplement: S1 File — Supplementary materials, including Tables S1 to S5 and experimental instructions for the stereotype threat and no-threat conditions. (DOCX) [file pone.0215050.s001.docx]

**Supplementary Materials for**

## Does social context impact metacognition?

## Evidence from stereotype threat in a visual search task

Thibault Gajdos^1^, Isabelle Régner^1^, Pascal Huguet^2^, Marine Hainguerlot^3^, Jean-Christophe Vergnaud^3^, Jérôme Sackur^4,5^, and Vincent de Gardelle^6^

^1^Aix Marseille Univ, CNRS, LPC, Marseille, France.
^2^Université Clermont Auvergne, CNRS, LAPSCO, Clermont-Ferrand, France.
^3^Centre d’Economie de la Sorbonne, CNRS UMR 8174, Paris, France.
^4^Laboratoire de Sciences Cognitives et Psycholinguistique (ENS, CNRS, EHESS), PSL Research University, Paris, France.
^5^Ecole Polytechnique, Palaiseau, France.
^6^CNRS and Paris School of Economics, Paris, France.

Table A: Mean accuracy scores and median response times.

|  |  |  |  | Accuracy | | Median response times | |
| --- | --- | --- | --- | --- | --- | --- | --- |
| Sex | Stereotype | Identification | Frame | *M* | *SD* | *M* | *SD* |
| Women | No-threat | Low | Gain | 0.90 | 0.091 | 1.1 | 0.39 |
|  |  |  | Loss | 0.89 | 0.098 | 1.1 | 0.36 |
|  |  | High | Gain | 0.88 | 0.108 | 1.3 | 0.40 |
|  |  |  | Loss | 0.87 | 0.120 | 1.3 | 0.46 |
|  | Threat | Low | Gain | 0.87 | 0.132 | 1.2 | 0.41 |
|  |  |  | Loss | 0.87 | 0.143 | 1.2 | 0.44 |
|  |  | High | Gain | 0.87 | 0.125 | 1.1 | 0.34 |
|  |  |  | Loss | 0.87 | 0.127 | 1.1 | 0.36 |
| Men | No-threat | Low | Gain | 0.85 | 0.125 | 1.1 | 0.41 |
|  |  |  | Loss | 0.87 | 0.113 | 1.2 | 0.40 |
|  |  | High | Gain | 0.89 | 0.113 | 1.3 | 0.46 |
|  |  |  | Loss | 0.88 | 0.125 | 1.3 | 0.48 |
|  | Threat | Low | Gain | 0.87 | 0.112 | 1.1 | 0.37 |
|  |  |  | Loss | 0.90 | 0.100 | 1.1 | 0.33 |
|  |  | High | Gain | 0.88 | 0.101 | 1.2 | 0.50 |
|  |  |  | Loss | 0.88 | 0.103 | 1.3 | 0.50 |

Table B: Regression analysis of response accuracy

| ACCURACY | *β* | se | F | p |  |
| --- | --- | --- | --- | --- | --- |
| target type | 0.0223 | 0.0054 | 252.4637 | <0.0001 | *** |
| set size | -0.0065 | 0.0004 | 195.2621 | <0.0001 | *** |
| stereotype | 0.0038 | 0.0057 | 0.4589 | 0.4995 |  |
| sex | 0.0033 | 0.0057 | 0.2350 | 0.6287 |  |
| frame | -0.0006 | 0.0018 | 0.1021 | 0.7499 |  |
| identification | 0.0007 | 0.0057 | 0.0079 | 0.9294 |  |
| target type × set size | -0.0051 | 0.0004 | 169.9470 | <0.0001 | *** |
| stereotype × sex | 0.0012 | 0.0057 | 0.0319 | 0.8587 |  |
| stereotype × frame | 0.0017 | 0.0018 | 0.8049 | 0.3715 |  |
| sex × frame | -0.0040 | 0.0018 | 5.0696 | 0.0262 | * |
| stereotype × identification | -0.0021 | 0.0057 | 0.1381 | 0.7108 |  |
| sex × identification | -0.0045 | 0.0057 | 0.6740 | 0.4133 |  |
| frame × identification | -0.0023 | 0.0018 | 1.5984 | 0.2086 |  |
| stereotype × sex × frame | -0.0004 | 0.0018 | 0.0435 | 0.8352 |  |
| stereotype × sex × identification | -0.0029 | 0.0057 | 0.2391 | 0.6258 |  |
| stereotype × frame × identification | -0.0003 | 0.0018 | 0.0209 | 0.8852 |  |
| sex × frame × identification | -0.0041 | 0.0018 | 4.7952 | 0.0305 | * |
| stereotype × sex × frame × identification | 0.0002 | 0.0018 | 0.0097 | 0.9217 |  |

Note: Predictors were coded as follows– target type: L=1, X=-1; frame: gain =1, loss=-1; stereotype: no-threat=1, threat=-1; sex: male=1,female=-1; identification: low=1, high=-1. **p < .*05; ***p < .*01: ****p < .*001

Table C: Regression analysis of median response times for correct responses

| RTs | *β* | se | F | p |  |
| --- | --- | --- | --- | --- | --- |
| target type | 0.0728 | 0.0107 | 589.9205 | <0.0001 | *** |
| set size | 0.0193 | 0.0009 | 299.2225 | <0.0001 | *** |
| stereotype | 0.0149 | 0.0235 | 0.3305 | 0.5665 |  |
| sex | 0.0029 | 0.0235 | 0.0009 | 0.9758 |  |
| frame | -0.0066 | 0.0088 | 0.5880 | 0.4447 |  |
| identification | -0.0306 | 0.0235 | 1.5904 | 0.2098 |  |
| target type × set size | 0.0120 | 0.0008 | 231.2575 | <0.0001 | *** |
| stereotype × sex | -0.0021 | 0.0235 | 0.0126 | 0.9108 |  |
| stereotype × frame | 0.0046 | 0.0084 | 0.2775 | 0.5993 |  |
| sex × frame | -0.0051 | 0.0084 | 0.3070 | 0.5806 |  |
| stereotype × identification | -0.0365 | 0.0235 | 2.4410 | 0.1209 |  |
| sex × identification | -0.0293 | 0.0235 | 1.1920 | 0.2772 |  |
| frame × identification | 0.0018 | 0.0084 | 0.0428 | 0.8365 |  |
| stereotype × sex × frame | -0.0073 | 0.0084 | 0.7849 | 0.3775 |  |
| stereotype × sex × identification | 0.0351 | 0.0235 | 1.9362 | 0.1667 |  |
| stereotype × frame × identification | -0.0052 | 0.0084 | 0.3748 | 0.5416 |  |
| sex × frame × identification | 0.0109 | 0.0084 | 1.6136 | 0.2065 |  |
| stereotype × sex × frame × identification | -0.0063 | 0.0084 | 0.5401 | 0.4638 |  |

Note: Predictors were coded as follows– target type: L=1, X=-1; frame: gain =1, loss=-1; stereotype: no-threat=1, threat=-1; sex: men=1, women=-1; identification: low=1, high=-1. **p < .*05; ***p < .*01: ****p < .*001

Table D: Regression analysis of Brier score

| BRIER SCORE | *β* | se | F | p |  |
| --- | --- | --- | --- | --- | --- |
| target type | -0.0061 | 0.0061 | 249.7997 | <0.0001 | *** |
| set size | 0.0061 | 0.0005 | 95.1495 | <0.0001 | *** |
| stereotype | 0.0041 | 0.0104 | 0.2175 | 0.6419 |  |
| sex | -0.0134 | 0.0104 | 0.5949 | 0.4421 |  |
| frame | -0.0136 | 0.0054 | 7.0953 | 0.0088 | ** |
| identification | 0.0095 | 0.0104 | 0.3454 | 0.5579 |  |
| target type × set size | 0.0049 | 0.0004 | 124.1772 | <0.0001 | *** |
| stereotype × sex | 0.0131 | 0.0104 | 0.2581 | 0.6124 |  |
| stereotype × frame | 0.0028 | 0.0053 | 0.1569 | 0.6927 |  |
| sex × frame | 0.0108 | 0.0053 | 4.2444 | 0.0416 | * |
| stereotype × identification | -0.0100 | 0.0104 | 1.7481 | 0.1887 |  |
| sex × identification | -0.0147 | 0.0104 | 1.0746 | 0.3021 |  |
| frame × identification | -0.0064 | 0.0053 | 1.3204 | 0.2529 |  |
| stereotype × sex × frame | -0.0158 | 0.0053 | 8.0650 | 0.0053 | ** |
| stereotype × sex × identification | 0.0088 | 0.0104 | 2.0737 | 0.1525 |  |
| stereotype × frame × identification | -0.0069 | 0.0053 | 1.5831 | 0.2108 |  |
| sex × frame × identification | 0.0075 | 0.0053 | 1.9566 | 0.1645 |  |
| stereotype × sex × frame × identification | 0.0112 | 0.0053 | 4.2507 | 0.0414 | * |

Note: Predictors were coded as follows– target type: L=1, X=-1; frame: gain =1, loss=-1; stereotype: no-threat=1, threat=-1; sex: men=1,women=-1; identification: low=1, high=-1. **p < .*05; ***p < .*01: ****p < .*001

Table E: Regression analysis of SNSI error

| SNSI ERROR | *β* | se | F | p |  |
| --- | --- | --- | --- | --- | --- |
| target type | 0.1014 | 0.0290 | 17.8721 | <0.0001 | *** |
| set size | 0.0000 | 0.0012 | 0.0000 | 1.0000 |  |
| stereotype | 0.0806 | 0.0506 | 2.2047 | 0.1403 |  |
| sex | 0.0099 | 0.0506 | 0.0222 | 0.8818 |  |
| frame | 0.0002 | 0.0228 | 0.0029 | 0.9572 |  |
| identification | -0.0246 | 0.0506 | 0.2218 | 0.6386 |  |
| target type × set size | 0.0000 | 0.0012 | 0.0000 | 1.0000 |  |
| stereotype × sex | -0.0519 | 0.0506 | 0.9605 | 0.3291 |  |
| stereotype × frame | -0.0130 | 0.0228 | 0.3526 | 0.5538 |  |
| sex × frame | 0.0093 | 0.0228 | 0.1900 | 0.6637 |  |
| stereotype × identification | -0.1082 | 0.0506 | 4.3984 | 0.0381 | * |
| sex × identification | -0.0128 | 0.0506 | 0.0766 | 0.7825 |  |
| frame × identification | -0.0021 | 0.0228 | 0.0038 | 0.9508 |  |
| stereotype × sex × frame | 0.0067 | 0.0228 | 0.1348 | 0.7142 |  |
| stereotype × sex × identification | 0.0721 | 0.0506 | 1.4873 | 0.2251 |  |
| stereotype × frame × identification | 0.0045 | 0.0228 | 0.0450 | 0.8324 |  |
| sex × frame × identification | 0.0101 | 0.0228 | 0.1961 | 0.6587 |  |
| stereotype × sex × frame × identification | 0.0473 | 0.0228 | 4.0827 | 0.0456 | * |

Note: Predictors were coded as follows– target type: L=1, X=-1; frame: gain =1, loss=-1; stereotype: no-threat=1, threat=-1; sex: men=1,women=-1; identification: low=1, high=-1. **p < .*05; ***p < .*01: ****p < .*001

**Exact instructions for Participants**

**Stereotype threat condition** : "This study tests the differences between men and women in the field of visuospatial skills that play a key role in geometry. For this, you will take a computer-based test that assesses your ability to quickly and correctly detect a visual target mixed with a set of distractors. Previous research show differences in performance between men and women on this test. "

• Focus on gains : "At the end of the test your score will be calculated as follows: 2 pts for each correct answer provided it is provided quickly, and zero point for each error. "

• Focus on losses : "At the end of the test your score will be calculated as follows: 1 point for each correct answer provided it is provided quickly, and minus 3 points for each error. "

**Control condition** : "This study tests the differences between men and women in the field of visuospatial skills that play a key role in geometry. For this, you will take a computer-based test that assesses your ability to quickly and correctly detect a visual target mixed with a set of distractors. Previous research shows that on this test there is no difference in performance between men and women. Men and women get the same performance. "

• Focus on gains : "At the end of the test your score will be calculated as follows: 2 pts for each correct answer provided it is provided quickly, and zero point for each error. "

• Focus on losses : "At the end of the test your score will be calculated as follows: 1 point for each correct answer provided it is provided quickly, and minus 3 points for each error. "
